# P-1830. Functional Status in Liver Transplant Recipients Receiving Grafts from HCV-Viremic versus HCV-Negative Donors: A Retrospective Analysis of the UNOS Database

**DOI:** 10.1093/ofid/ofaf695.1999

**Published:** 2026-01-11

**Authors:** Konstantinos Ouranos, Evangelia K Mylona, Fadi Shehadeh, R Mark Ghobrial, Eleftherios Mylonakis

**Affiliations:** Houston Methodist Hospital, Houston, Texas; Houston Methodist Hospital, Houston, Texas; Houston Methodist Research Institute, Houston, TX, Houston, Texas; Houston Methodist Hospital, Houston, Texas; Houston Methodist Hospital, Houston, TX, Houston, Texas

## Abstract

**Background:**

Use of HCV-viremic donors in liver transplantation (LT) has increased with the advent of direct-acting antiviral (DAA) therapy. While patient and graft survival rates are comparable to LT from HCV-negative donors, the impact of receiving HCV-viremic grafts on quality of life metrics, such as functional status, has not been studied.

**Table 1:**
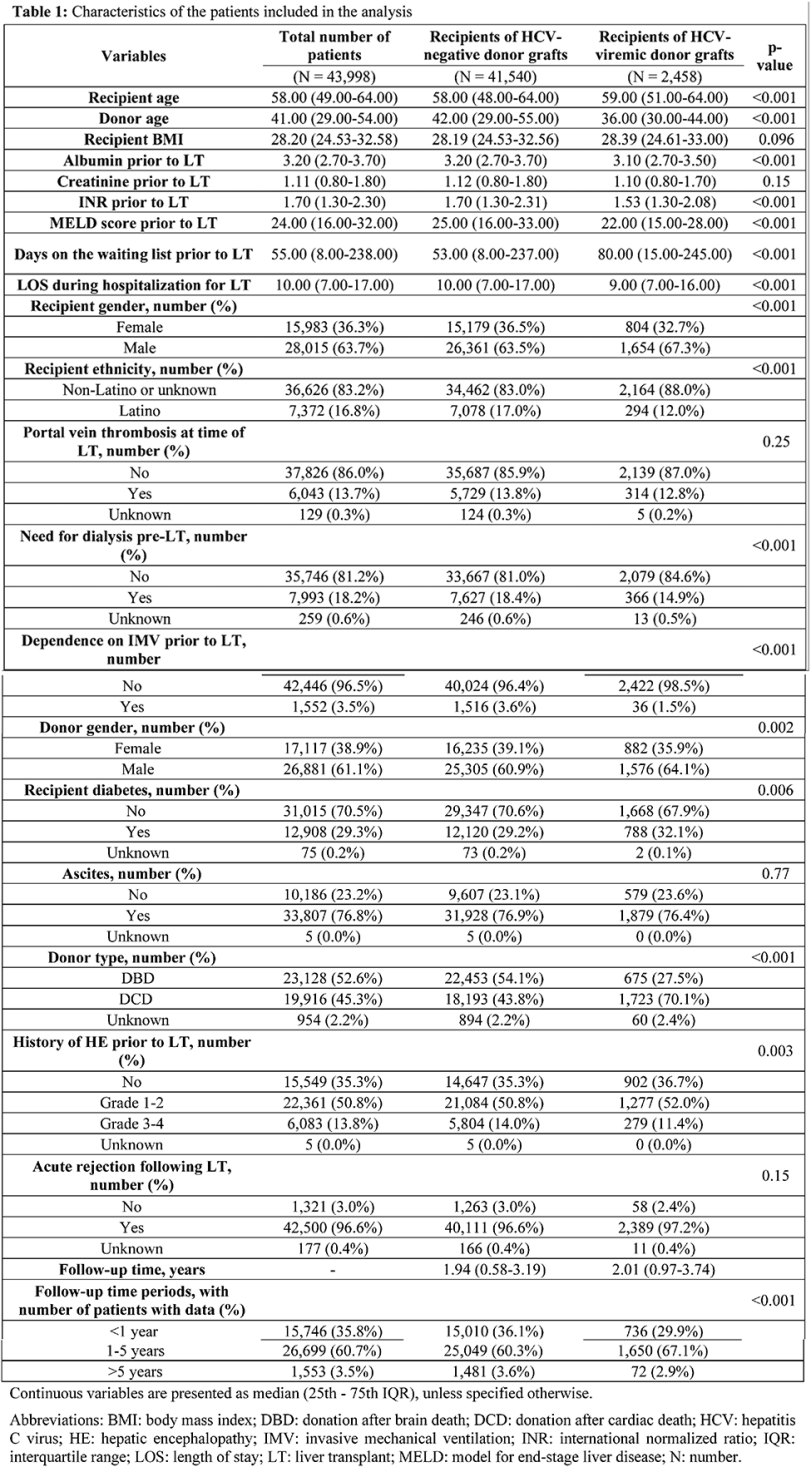
Characteristics of the patients included in the analysis Continuous variables are presented as median (25th - 75th IQR), unless specified otherwise. Abbreviations: BMI: body mass index; DBD: donation after brain death; DCD: donation after cardiac death; HCV: hepatitis C virus; HE: hepatic encephalopathy; IMV: invasive mechanical ventilation; INR: international normalized ratio; IQR: interquartile range; LOS: length of stay; LT: liver transplant; MELD: model for end-stage liver disease; N: number.

**Table 2:**
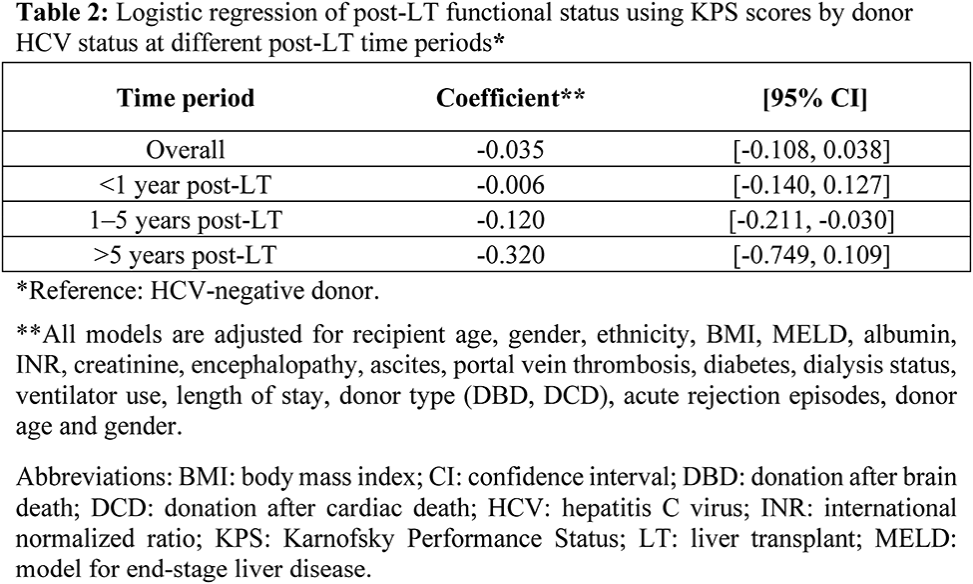
Logistic regression of post-LT functional status using KPS scores by donor HCV status at different post-LT time periods* *Reference: HCV-negative donor. **All models are adjusted for recipient age, gender, ethnicity, BMI, MELD, albumin, INR, creatinine, encephalopathy, ascites, portal vein thrombosis, diabetes, dialysis status, ventilator use, length of stay, donor type (DBD, DCD), acute rejection episodes, donor age and gender. Abbreviations: BMI: body mass index; CI: confidence interval; DBD: donation after brain death; DCD: donation after cardiac death; HCV: hepatitis C virus; INR: international normalized ratio; KPS: Karnofsky Performance Status; LT: liver transplant; MELD: model for end-stage liver disease.

**Methods:**

We performed a retrospective analysis of the UNOS database to assess post-LT functional status in adults receiving grafts from HCV-viremic vs HCV-negative donors after DAA therapy approval. We assessed post-LT functional status using the Karnofsky Performance Status (KPS) scale (0–100, with lower scores indicating greater disability). KPS scores were analyzed for all patients and within three follow-up periods: < 1 year, 1–5 years, and >5 years post-LT. We used ordered logistic regression to model the odds of higher KPS score by donor HCV status, adjusting for key recipient, donor, and transplant factors. Analyses were performed with Stata 17.0.

**Results:**

Our analysis included 43,998 LT recipients, of whom 2,458 received HCV-positive and 41,540 received HCV-negative grafts. HCV-positive graft recipients tended to be older, male, have younger donors, lower MELD scores, longer waitlist times, diabetes, and grafts from donors after circulatory death. Median follow-up times for recipients of HCV-viremic versus HCV-negative donors was 2.01 and 1.94 years, respectively. Using logistic regression models, we found that donor HCV status was not significantly associated with differences in KPS scores after LT (coefficient, -0.035; 95% CI: -0.108, 0.038). Analyses at the three pre-defined time periods showed that while recipients of HCV-viremic grafts had statistically lower odds of better functional status at 1–5 years post-LT compared to recipients of HCV-negative grafts (coefficient, -0.120, 95% CI: -0.211, -0.030), the difference is small and unlikely to be clinically meaningful. HCV status was not significantly associated with differences in KPS scores after LT at < 1 and >5 years post-LT.

**Conclusion:**

Our analysis shows that donor HCV status does not affect post-LT functional status when accounting for key recipient, donor, and transplant factors such as MELD score, demographics, and comorbidities.

**Disclosures:**

Eleftherios Mylonakis, MD, PhD, Chemic Labs/Koda Therapeutics, LLC: Grant/Research Support|Lumen: DSMB|NIH/NIAID: Grant/Research Support|Sciclone: Grant/Research Support|Shionogi: Advisor/Consultant|Synexis: Clinical trial

